# Supplementary figures and images for: Immune alterations in subacute sclerosing panencephalitis reflect an incompetent response to eliminate the measles virus
Source: PLoS One. 2021 Jan 7;16(1):e0245077. doi: 10.1371/journal.pone.0245077 (PMC7790413; doi:10.1371/journal.pone.0245077)

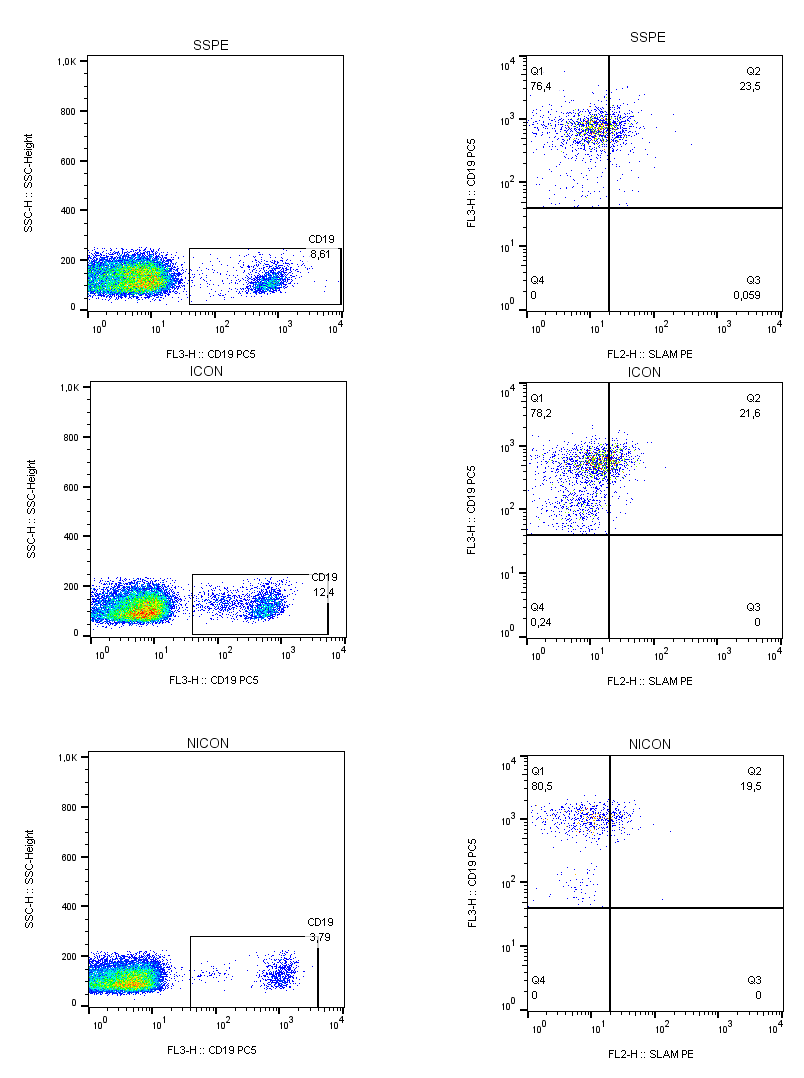

Supplement: S1 Fig — SLAM expression on CD19+ cells in a subacute sclerosing panencephalitis patient (SSPE) and in controls with inflammatory diseases (ICON) or non-inflammatory diseases (NICON) are shown. (TIF) [file pone.0245077.s001.tif]

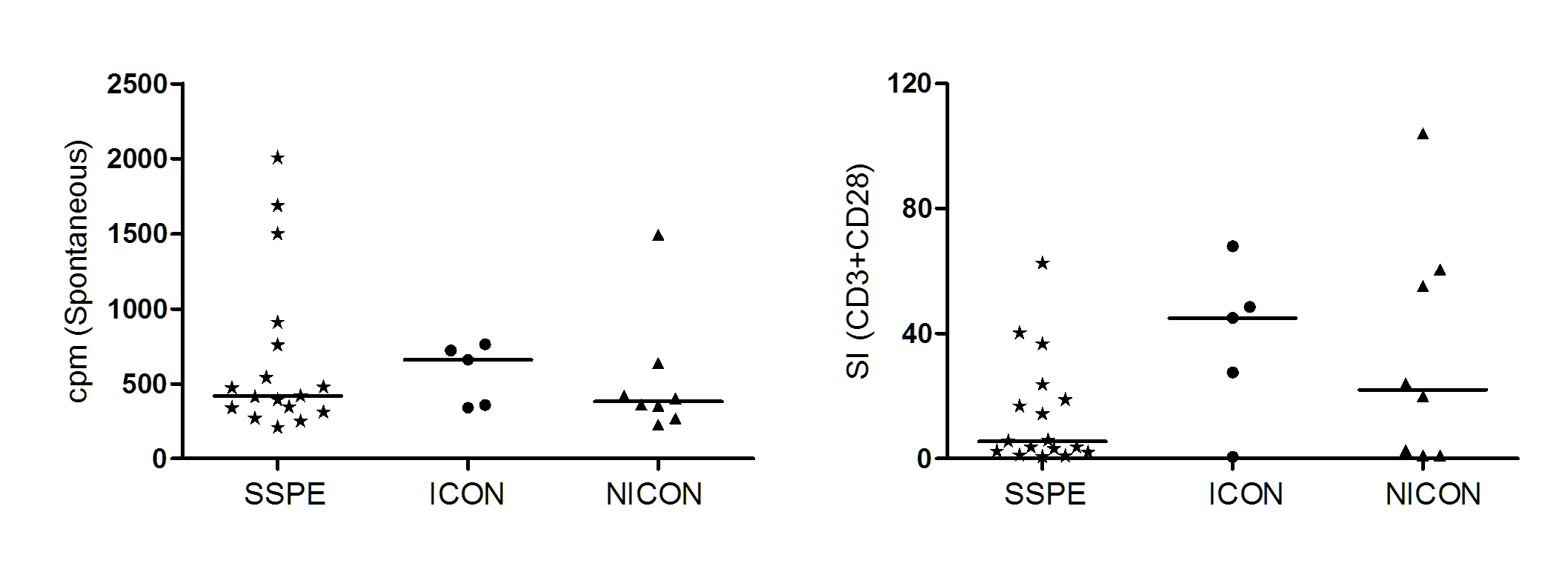

Supplement: S2 Fig — Spontaneous and CD3+CD28 induced proliferative responses of T cells in subacute sclerosing panencephalitis (SSPE) patients and in controls with inflammatory diseases (ICON) and non-inflammatory diseases (NICON) are shown. Cpm: Counts per minute, SI: Stimulation index (SI = Induced cpm / Spontaneous cpm). (TIF) [file pone.0245077.s002.tif]

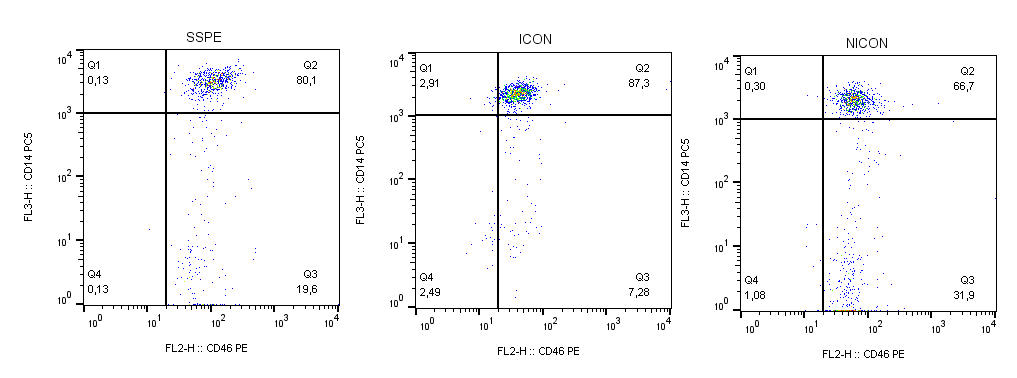

Supplement: S3 Fig — CD14+CD46+ monocytes in a subacute sclerosing panencephalitis patient (SSPE), controls with inflammatory diseases (ICON) and with non-inflammatory diseases (NICON) are shown. (TIF) [file pone.0245077.s003.tif]

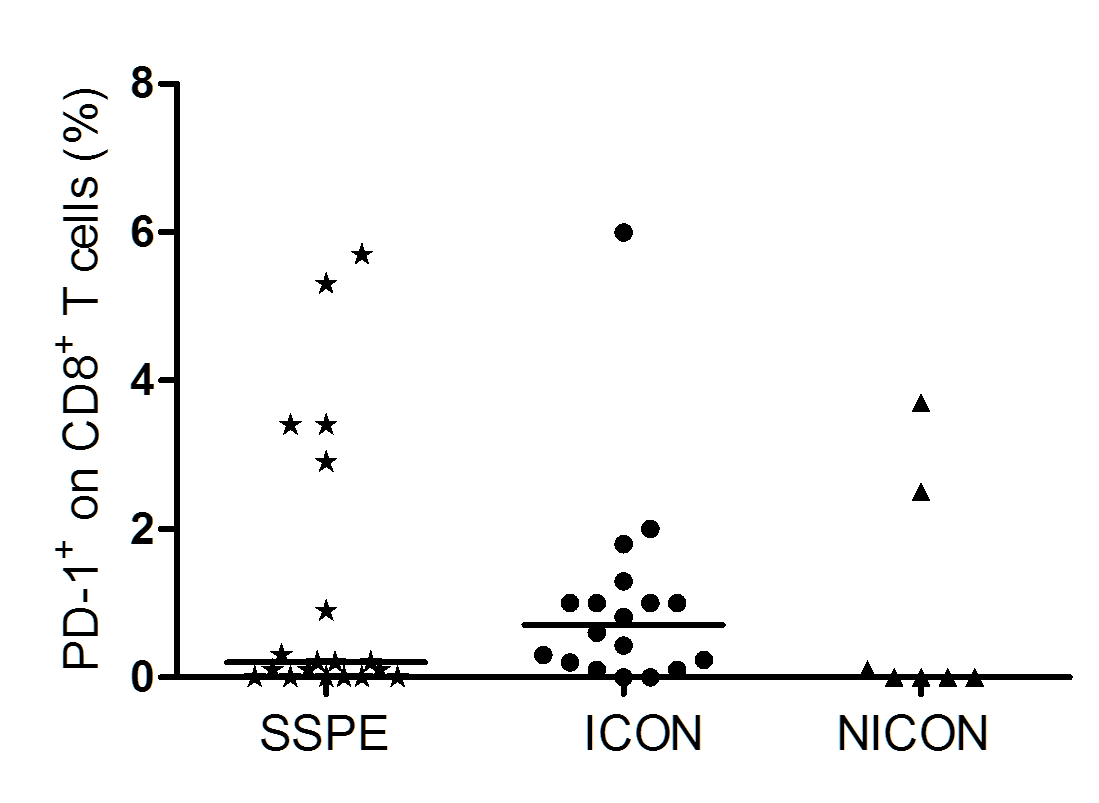

Supplement: S4 Fig — PD-1 on CD8+ T cells in a subacute sclerosing panencephalitis patient (SSPE), controls with inflammatory diseases (ICON) and with non-inflammatory diseases (NICON) are shown. Horizontal lines depict median values. (TIF) [file pone.0245077.s004.tif]
