# Supplementary material for: Immune alterations in subacute sclerosing panencephalitis reflect an incompetent response to eliminate the measles virus
Source: PLoS One. 2021 Jan 7;16(1):e0245077. doi: 10.1371/journal.pone.0245077 (PMC7790413; doi:10.1371/journal.pone.0245077)
Supplement: S1 Data — (PDF) [file pone.0245077.s005.pdf]

SSPE DNA #	SSPE FENO CD4+	SSPE FENO CD8+	SSPE FENO CD19+	SSPE FENO CD3+	SSPE FENO CD14+	IKON DNA #	IKON FENO CD4+	IKON FENO CD8+
**2315**	42,5	25,5		70		**3573**	29,5	24,4
**2325**				58	72	**3574**	28,2	28,4
**2379**	30,1	39,5		68,5		**3575**	44,5	33
**6707**	41,9	16,7		62	63,6	**3576**	53,7	19,6
**6712**	25,9	26,3		51,5	25,6	**3577**	46,1	28,1
**6719**	35,1	37,2		67	32,4	**3578**	47,7	27,3
**6729**		15,9		41,8	80,5	**3579**	53,5	32
**6734**	32,2	33,9		68,3	74,8	**3580**	33,9	41,9
**6735**	25,8	24,4		56,6	85,6	**3581**	47,1	25,2
**6750**	30,5	21,7		61,5	29,2	**3582**	32,4	29,2
**6751**	29,6	23,6		59,2	35,4	**3612**		
**6752**	30,5	25		64,7	62,7	**3613**		
**6753**	29,9	25		66	7,4	**3678**	45,3	26,3
**6754**	11,6	25,3			74,2	**3679**	33,5	30,6
**6760**	36,5	32,9			90,7	**3683**	42,6	27,3
**6762**	16,2	19,4		50,9	74	**3684**	33,9	24,1
**6763**	56	44,6		72,3	78,6	**3685**	20,6	31,2
**6764**	27,4	38,3		68,4	60,3	**5831**	35,3	24,5
**6765**	21	23,9		50,1	93,6	**5833**	21,8	27,5
**6766**	42	28		71,3	67	**6714**	40,5	25
**6767**	15,3	45		65,5	81,2	**6715**	42	30
**6768**	31,9	36,6		60,5	59,6	**6717**	30,5	22,7
**6769**	27,9	21,7		52,5	52,8	**6720**	39,2	44,5
**6776**	34,4	26,1		66,9	88,2	**6723**	18,7	34,8
**6777**	25,3	20,4		47,9	63,5	**6724**	22	20
**6786**	18,1	12,2		73,3	85,2	**6725**	36,5	22,5
**6787**	16,6	18,7		50,7	64,8	**6727**	30,7	27,6
**6788**	27,5	23		53,7	74,3	**6731**		29,2
**6789**	36,6	23,4		64	32,1	**6732**		24,1
**6792**	21	15		34,1	25	**6736**	34,1	27,6
**6793**	30	26,4		60,5	62,7	**6737**	25,8	36,5
**6794**	39,8	16,1		59,3	59,8	**6738**	28	33,5
**6796**	43,6	22,8		67,2	81,6	**6739**	20,6	7,9
**6797**				65	92	**6740**	36	24,5
**6798**				42,4	3,9	**6741**	11,8	18,2
**7801**				49,3	5,2	**6742**	37,7	22
**7803**	11,4	29,7		33,5	82,3	**6761**	36,3	15,8
**7805**	24,3	38,9		67,7	89,3	**6770**	16	17,6
**7806**	40,9	32,3		74,4	72,7	**6771**	31	25
**7809**	62,8			66,5	95	**6772**	13	17,7
**7810**	26,2	40,6		76,4	94,5	**6778**	25	34,3
**7813**	34,5	35		70,4	93	**6783**	31,7	29,9
**7818**	38,6	41,8		79,2	78,6	**6784**	40,6	16,2
**7822**	41,3	24,5				**6790**	23,2	14,3
**7823**	45,9	9				**6791**	18,4	11
**7826**	43,3	22,1				**6800**		

**7828**	35,8	25,1			
**7829**	34,6	27,9		66,5	
**7830**	49,9	33,8			
**7831**	26,5	38,3			
**7832**	13,6	22,2			
**7833**	41,4	35,3			
**7835**					
**7837**	57,9	21,9			29,6
**7840**	23,7	19,6			
**7841**	40	19,5			64,4
**7844**	47,1	22,2			74
**7846**	25	38,5			66,6
**7849**	33,3	36,7			73,7
**7850**	40,9	17			79,8
**7853**	41,5	22			69,4
**7854**	33,4	24,6			86
**7856**	29,7	12,5			92,7
**7858**	27,9	21,8			71,6
**7859**	31,5	35,3			
**7861**	12,5	26,2			61,1
**7863**	16,2	13,3			59,6
**7866**	32,3	30,7	17,3		
**7868**	19,3	22,2	22,2		
**7869**	18,1	22,1	18,1		
**7872**	34,9	26,9	12,9		
**7873**	32,2	35,1	10,2		
**7874**	24,8	22,5	6,8		
**7876**	29,1	29,2	14,1		
**7879**	29,8	39,7	2,4		
**7880**	35	23,5	3,1		
**7883**	33	24,8	11,2		
**7884**	28,6	41,2	11,6		
**7886**	24,1				
**7888**	23,4	18,8	25,7		
**7889**	27,4	37,1	6,9		
**7891**	44,7	29,9	12,2		
**7892**	41,3	29,4	5,9		
**7893**	34,3	35,4			
**7899**	32,4	30,4	12,5		
**8042**	35	34,7	9	67,4	88,5
**8054**	48,1	33,9	2		97,4
**8056**	54,3	21,8	18		
**8057**	51,5	29,1	13		
**8061**	48,9	32,2	12	82,7	99,1
**8065**	44,6	28,4	9	79,4	91,3
**8066**	36,8	37,9	7	71,4	92,5
**8067**	46,3	27,4	14	70,6	91,4
**8068**	27,1	30,6	19	63,8	88
**8069**	21,9	7,5	3	44,6	
**8069**	50,84	12,77	5,15	76,1	2,42

**7802**		
**7804**		
**7819**	49,8	33,7
**7842**	37,6	20,3
**7848**	42,8	25,3
**7852**	42,6	21
**7855**	34	16
**7857**	41,4	47,9
**7860**	46	31,5
**7862**	26,7	13,8
**7865**	34,5	26,4
**7867**	41,2	24,2
**7871**	23,8	24,9
**8215**	49,5	22,2
**8216**	26,8	47,2
**8224**	37	20,2
**8225**	30,1	34,2
**8227**	43,4	23
**8228**	47,6	28,4
**8234**	46,3	23,3
**8238**	49,5	28,5
**8280**	30,7	20,1
**8281**	28,3	33,4
**8282**	21,3	36,6
**8283**	39,2	23,1

**8070**	6,6	4,2	1	12,7	8,3
**8070**	41,82	25,79	5,68	15,49	7,91

IKON FENO CD19+	IKON FENO CD3+	IKON FENO CD14+	NIKON DNA #	NIKON FENO CD4+	NIKON FENO CD8+	NIKON FENO CD19+	NIKON FENO CD3+	NIKON FENO CD14+
13,9	64,1		**3559**	28,8	45	4,1	67,7	65,39
12,5	0,4	95,1	**3560**	27,8	49,5	10,5	64	95,4
13,2	71,7	52,9	**3565**					
6,1	75,29	93,84	**3614**					
6,3	77,17	85,48	**4344**	49,1	23,4		76,5	77
	76,6	96,9	**4379**	37,8	49		78,6	70,5
2,6	82,5	94,4	**4382**					
6,5	77,8	98,8	**5832**	35,5	33,2	6,4		
12,2	67,2	78,9	**5834**	29,5	43,6	7		
10,2	66,5	90,6	**6716**	41,9	31,8		76,5	91
			**6721**	41,6	30			73,4
			**6722**	35,1	34,3		68,9	67,3
11,5	71	70,6	**6726**	48,9	24,1		79,6	70
17,5	61,7	94,7	**6728**	18	27		51,4	73
6,4	69	90	**6730**		18,9		65,1	84,9
7,3	56,8	95,2	**6733**		18		64,2	85
6,1	65	84,5	**6743**	12,5	29,5		77,7	
13,6			**6744**		17,9			96
8,7			**6759**	45,6	26,8		74,5	48,6
	66,2	87,2	**6779**	36,4	25,8		62	88,2
	70	93,9	**6780**	38,8	29,3		72,9	79
	61,5	84,2	**6781**	26,6	24		49,8	75,8
	81,8	73	**6782**	17,5	23,7		80,5	76,8
	61,2	76,5	**6785**	32	17		77,6	69
	46,1	72,5	**6799**				76,1	12,1
	68,8	61,7	**7807**	59,2			46	57,5
	66,7	50,5	**7811**					
	71,3	72	**7812**	32,9	21,5		68	60,4
	67,2	76	**7814**					
	59,2	85	**7815**					
	54	87	**7820**	33,8	52,8		77,8	66,4
	64	79,8	**7824**	44,9	22,5			
	58	90,2	**7825**	23,7	31,4			
	49,4	89	**7827**	38,2	22			
	80		**7834**	34,1	32			
	71	91,6	**7845**		36,6			67,6
	60,6	72,9	**7847**	47,9	21,7			48,9
	43,5	29,5	**7851**	35,5	27,1			48,4
	76,4	65,4	**7864**	38,3	28,3			
	16	75	**7870**	36,3	15,7	13		
	47,7	82,7	**7885**	27,5	38,1	10,4		
	63,7	76,9	**7896**	57,9	28	3,8		
	77	66	**8060**	51,9	19,4	16	75,2	95,9
	40,9	46	**8071**	45,47	24,72	5,04	29,21	5,46
	46,6	41,3						

	75,7	79,1
	80,3	62,9
		81,4
		73
		41,9
		67,2
		77,4
		52,2
		68,8
11,2		
12,3		
		37,3
		49,9
		26,8
		42,4
		32,5
		13,1
10,1		
8,2		
12,8		
10,1		

DONÖR	SSPE SI (-) IgG1 SPON	SSPE SI CD3+CD28	SSPE (-) IgG1 SPON-IL-2	SSPE CD3/CD28-IL-2	SSPE (-) IgG1 SPON-IL-10	SSPE CD3/CD28-IL-10	SSPE (-) IgG1 SPON-IL-12p40	SSPE CD3/CD28-IL-12p40
**2325**			32,02	11,86				
**2385**			0	0	8,7	5,2	574,3	291,7
**2389**	2004,8	5,7	0	0	2	2,2	12,5	31,5
**2390**	1686	0,7	0	0	3,8	3,2	56,9	44,5
**2391**	252	3,8	0	0	4,6	8,3	40,6	30,8
**2392**			0	0	0,7	2,5	7,4	12,2
**2394**	209,5	2,4	0	38,5	1,7	0,3	7,7	6,1
**2396**	1499,5	14,3	0	0	1,5	6,3	254,3	646,5
**2397**			0	0	2,8	1,4	23,1	31,3
**2398**	909,7	40,3	0	108,9	45,7	212,1	851	720,3
**2399**	419,8	23,7	3,6	103,7	14,3	16,9	392,7	446,4
**2400**	539,8	5,9	1,3	111	4,3	8,3	165,2	310,1
**6701**	478,3	0,9	12	72,8	0,8	1	304,5	237,8
**6702**	411,7	1,1	83,8	72,8	0,8	1,6	330,9	225,2
**6703**	311,5	16,9	1035	244,3	0,5	1,1	233,8	196,3
**6704**	269	18,9	287,5	83,8	2,8	5,5	445,1	285,9
**6712**	393,5	36,7	428	183	0,3	1,8	225,2	208,5
**6729**	756,3	3,2	273,8	130,1	12,1	4,5	285,9	218,4
**6750**	344,3	62,5	9,8	6,7	2,7	16,5	332,6	551,7
**6751**	472	2	8	7,1	3	3,1	196,8	89,1
**6752**	339,3	3,7	10,7	8	0,2	0,3	222,5	79,6
**6797**								
**6798**			10	28,1	12,6	11,3	385,8	153,4
**7803**			5	18,7	30	141,3	1657,5	1687,7
**7805**			23,9	39,4	220,9	108,4	545,6	1459,1
**7806**			8,7	31,2	6,7	9	60,1	62,8
**7809**			33,3	13,1	706,5	246	888,7	446,4
**7810**			15	16,6	22,8	26,1	202,2	265,1
**7813**			8,1	40,7	44,1	127,8	1912,5	1900,2
**7818**			4,4	10,6	15,3	3,3	90,8	33,7

SSPE (-) IgG1 SPON-IL-12p70	SSPE CD3/CD28-IL-12p70	SSPE (-) IgG1 SPON-IFNg	SSPE CD3/CD28-IFNg	DONÖR	IKON SI (-) IgG1 SPON	IKON SI CD3+CD28	IKON (-) IgG1 SPON-IL-2	IKON CD3/CD28-IL-2
		1,6	235,3	**6723**	341,5	67,9	0	0
30,7	28,7			**6724**	658,7	0,7	0	0
6,9	9,1	0	0	**6727**	359,8	45,1	0	2,6
6	6,9	0,4	0	**6725**			7,6	9,4
6,6	7,5	0,1	0	**6742**	764,5	48,6	11,6	0,9
6,4	7,8	0	0,4	**6748**	721	27,6	7,1	9,8
8,3	14,2	0	0	**2386**			0	13,1
6	26,1	0	142,4	**2395**			10	8,7
7,7	8,8	0	0,1	**7802**				39
9,8	10,7	17,3	2069,4	**7804**			8,7	6,9
7,3	8,2	2,6	8,9	**7808**			8,7	14,4
6,2	9,2	1,3	16,4	**7816**			31,2	14,4
8,1	7,1	0,4	0,5	**7819**			61,7	8,7
5,7	7,3	0,4	1					
7	7,4	0,9	4,2					
9,4	5,4	0,9	32					
15,4	14,8	1,1	3,4					
6	13,2	0,6	0,6					
14	14,3	0,3	2,9					
13,9	12,9	0,2	1,3					
13,6	13,7	0,4	3,2					
		4,3	9,4					
20,8	19,9							
45,3	61,8	12,5	1777,8					
0,4	22,9	2,2	901					
0,7	1,8	2,3	14,5					
2,2	2,7	2,6	2355,9					
0	1,6	19,9	1496,5					
96,5	232,9	779,5	1352,4					
0	0	1,6	1,6					

IKON (-) IgG1 SPON-IL-10	IKON CD3/CD28-IL-10	IKON(-) IgG1 SPON-IL-12p40	IKON CD3/CD28-IL-12p40	IKON (-) IgG1 SPON-IL-12p70	IKON CD3/CD28-IL-12p70	IKON (-) IgG1 SPON-IFNg	IKON CD3/CD28-IFNg	DONÖR
13,1	19,2	491,7	686,6	11,4	17,7	0	939,2	**6716**
14,3	20,3	402,1	504,7	13,5	17,2			**6718**
32,4	40,2	56,2	78,2	10,7	13,4	0,1	13,7	**6722**
29,8	35,5	451,6	283,7	10,6	12,5	0,5	91,2	**6726**
11,8	64,8	498,3	538,8	11,3	14,4	5,9	1058,2	**6744**
4,6	7,7	445,1	354,7	11,9	16,3			**6747**
1,7	4,8	13,9	68,8	4,8	11,4	5	3,4	**6728**
2,2	19,4	30,1	47,6	4,2	14,8	2	71,6	**6743**
348,7	698,6		1201		24,3		711,8	**6745**
118,1	61,8	836,3	1354,4	2,6	17,7	2,6	156	**6746**
204,1	201,8	50,6	46	0,9	0	3,2		**7807**
138,9	900	2200	1863,9	73,4	29,4	1033	245,2	**7811**
108,2	66,4	661,4	861	21,7	15,2	152,3	2048,6	**7814**
								**7815**
								**7817**
								**7820**

NIKON SI (-) IgG1 SPON	NIKON SI CD3+CD28	NIKON (-) IgG1 SPON-IL-2	NIKON CD3/CD28-IL-2	NIKON (-) IgG1 SPON-IL-10	NIKON CD3/CD28-IL-10	NIKON (-) IgG1 SPON-IL-12p40	NIKON CD3/CD28-IL-12p40	NIKON (-) IgG1 SPON-IL-12p70
		0	0	87,3	42,1	249,6	124,4	4,1
420	1	0	0	40,9	22	465,6	150,7	6
1491,8	24,1	0	0	38,3	29,7	296	187,8	4,2
636	55,2	0	0	17,9	54,2	193,5	289	10,8
403,8	60,6	0	0	8,7	18,4	311,2	313,5	14,3
		0	0	4,4	3,2	717,8	453,7	15,2
361	20	5,8	9,4	48	55,9	283,3	168,8	10,7
268,8	104	4,9	0	4,8	32,2	370,2	227,9	12,2
226,7	1	0	0	1,2	0,3	13,6	3,7	8,5
353	2,8	0	0	5,1	2,1	277,2	127,3	6
		6,9	5,6	588,6	159,2	1545,4	1093,2	7,5
		11,2	33,7	130,6	73,8	197,7	150,7	0,5
		13,7	15	94,1	183,4	1011,9	1119,7	4,4
		13,1	7,5	71,8	36,9	442,9	390,6	6,9
		32,4	44,8	109,7	273	2200	2200	95,2
		47,3	6,9	60,4	105,6	433	1154,2	0

NIKON CD3/CD28-IL-12p70	NIKON (-) IgG1 SPON-IFNg	NIKON CD3/CD28-IFNg
5	0	0,2
6,3	1,5	0
11,1	0	0
14	0	1185,8
14,6	0	178,7
13	0	0
14,8	1,2	17,5
12,4	1,5	751,7
8,3	0,4	0,1
11,8	0,3	0,6
3,8	18,1	2574,7
1,4	1,9	572,9
9,1	40	2184
5,7	12,7	23,3
53,5	242,4	574,7
4,9	15,7	240,1

DNA # SSPE	(-) IL12P70 SSPE	SAC IL12P70 SSPE	(-) IFNG SSPE	SAC IFNG SSPE	(-) IL10 SSPE	SAC IL10 SSPE	(-) IL23 SSPE	SAC IL23 SSPE
**2397**	7,8	5	0,9	0			27,5	32,8
**6752**	18,5	17,5	0	0	16,2	17,5	22,6	23,3
**2382**	5,2	5,3	3,6	7,3	1,2	125,4		
**6704**	6,6	5,3	0	1,7	0	8,6	28,2	28,6
**2381**	5,9	0,6	0	0	5,4	11,6	44,4	28,6
**2379**	4,1	3,5	0	0	20,2	0		
**2380**	11,1	5,1	0	0	0	8,6	25,8	31,9
**6750**	15,6	14,7	0	0	24,7	37,6	19,6	22,3
**2399**	18,2	16,2	0,6	13,3	3,8	58,7	22,9	34,7
**2376**	2,7	2,2						
**2372**	3,8	2,9	0,1	0,2	14,5	13,1		
**2375**	3,8	5,1	0	1,8	15,3	43,2	31,1	27,8
**2394**	5,2	5,2	0	0	0	23,8	25,1	27,4
**6702**	19,3	16,7	0	12	0	0	44,4	139,8
**6701**	16,7	5,7	0	0	18,8	15,3	45,4	49,2
**6754**	17,8	17,1	0	0	4,6	29,9	46,3	39,4
**2391**	6,9	1,6	0	0				
**2374**	2,4	2,2	0	0	0	1,2		
**6703**	3,5	3,7	0	0,2	16,2	32,7	24,7	26,6
**2392**	3,9	11,8	0	0				
**2396**			0	59,4	25,6	27	31,1	33,6
**2304**	3,7	1,6					26,6	24,3
**6712**	8,4	7,4	0	0,2	23,8	55,9	17,7	24,3
**2400**	13,1	10,2	0	0	1,2	0	22,9	22,6
**2398**	4,9	3,6			22,9	39,6	27,4	33,2
**6729**	5,9	7,8	7,3	72,3	17,5	28,9	37,6	27,8
**2303**	7	6,5						

(-) IL12P40 SSPE	SAC IL12P40 SSPE	DNA # ICON	(-) IL12P70 ICON	SAC IL12P70 ICON	(-) IFNG ICON	SAC IFNG ICON	(-) IL10 ICON	SAC IL10 ICON
19,3	13,2	**6748**	17,6	17	0	0,5	16,2	20,6
69,7	209,6	**6724**	6,8	10,9	0	0	0	28,8
372,3	610,4	**6723**	10	10,1	0	0	11,6	25,3
180,2	176,7	**6742**	16	12,5	0,1	0,9	0	10,8
99,4	105,1	**6727**	6,9	8,8	0	0	35	262,8
48,6	21,1	**6725**	9,2	7,3	0	0	15,3	31,6
1,9	7,7							
145,3	466,7							
221	703,2							
9,3	47,3							
472,9	400,9							
76	111,4							
0	0							
32,9	163,3							
25,2	30,3							
79,1	277,6							
48,5	48,2							
32,6	41,2							
6,1	18,3							
0	0							
110,4	228,2							
13,4	195,7							
283,8	103,5							
387,7	619,3							

(-) IL23 ICON	SAC IL23 ICON	(-) IL12P40 ICON	SAC IL12P40 ICON	DNA # NICON1	(-) IL12P70 NICON1	SAC IL12P70 NICON1	(-) IFNG NICON1	SAC IFNG NICON1
20,3	27,5	211,6	344,2	**6747**	16,3	15,8	0	0
23,3	24,7	331,3	370	**6746**	18,4	16,9	0	0
33,9	29,7	414	328,7	**6744**	16,6	16,4	0	6,5
36,3	24	181,1	238	**6755**			0	0,2
27,5	26,4	57,3	208,4	**6757**			0	0,6
22,6	24	148,5	165,8	**6726**	5,9	9,7	0	1,4
				**6756**			0	0
				**6718**	11,8	7,1	0,1	14,3
				**6728**	7	7,1	0	0
				**6745**	16	16,4	0	0
				**6743**	7,8	4,3	0	0,5
				**6716**	12,2	9,8	0	0,6
				**6758**				
				**6722**	11,3	9,4	0	0,7
				**4382**	5,3	5,5	0	0
				**4377**	12,6	10,3	0	1,1
				**4381**	5,2	5,6	0	0
				**4385**	2,5	12,4	1,5	15,7
				**4359**	17,9	16,5	0	0
				**4383**	4,8	5,5	0	0
				**4379**	16,9	11,9		
				**4387**	14,1	12,8	0	3,1
				**4393**	5,2	4,7	0,6	0
				**4392**	5,5	6,5	0	0
				**4396**	5,4	5,9	0	0
				**4404**	5,3	6,9	0	0
				**4378**	15,3	15,2	0	0

(-) IL10 NICON1	SAC IL10 NICON1	(-) IL23 NICON1	SAC IL23 NICON1	(-) IL12P40 NICON1	SAC IL12P40 NICON1
18,4	40,6	26,8	22,9	904,3	4002,6
17,5	17,5	24	23,3	309,6	174,7
0	19,6	27,5	29,7	221,6	319,2
18,4	19,7	29,9	32,4	56,7	58,5
17,1	18,8	22,9	29,3	91,4	93,4
3	38,4	32,8	34,3	123,7	219,7
45,1	29,9	20,9	22,9	83,5	85,9
6,2	67,3	36,7	32,4	360,4	621,6
27,4	93,1	30,1	27,2	116,5	157
20,6	15,3	24,7	23,6	17,4	23,4
0	0	21,6	24	74	67,5
17,5	98,4	36,7	34,1	200,6	273,6
7	11,6	23,1	23,5		
1,2	14,5	32,4	30,7	87,2	136,9
		31,5	48,7	46,6	101,4
25,6	30,8	22,3	26,1	14,9	24,3
24,7	25,6	20,6	24,3	45,1	64
18,4	114,5	48,2	32,8	164,1	198,9
19,7	18,8	21,9	24,3	7,4	3,4
20,2	21,1	19,6	23,3	61,4	91
14,9	89,7	38,9	29	59,3	139,8
17,1	27,5	29,7	24,7	47,8	29,9
				0	0
14,1	40,6	22,9	28,6	115,7	236,4
14,1	38,1	36,2	29	0	8
15,8	24,7	25	27,9	54,9	107,4

DNA SSPE	CD4+DE CD4+ SLAM+ SSPE	CD8+DE CD8+ SLAM+ SSPE	CD8+ DE PD1+ SSPE	CD19+ DA SLAM+ SSPE	CD14+ CD46+ SSPE	DNA İKON	CD4+DE CD4+ SLAM+ İKON	CD8+DE CD8+ SLAM+ İKON
**7856**	1,95	1,61				**3678**	39,9	45,98
**7888**	0,58	0,28	0,08	0,07		**3679**	30,6	13,2
**7886**						**3684**	24,95	6,17
**2315**						**3685**	15,17	10,89
**2325**					24,3	**8228**	2,2	0,4
**2379**						**8238**	3,3	
**6707**					63,6	**3683**	17,46	8,14
**6712**					25,55	**6761**		
**6719**					32,4	**6771**		
**6729**					80,3	**6772**		
**6734**					22,3	**7857**		
**6735**					69,12	**7865**	1,3	14,32
**6750**					3,13	**7871**	3,2	0,03
**6751**					7,32	**6790**		
**6752**					62,2	**6791**		
**6753**					7,26	**7804**		
**6754**					75,6	**7848**	0,62	0
**6760**					90,7	**6714**		
**6762**					70,9	**6717**		
**6763**					78,6	**6723**		
**6764**					59,3	**6731**		
**6765**					91,01	**6732**		
**6766**					66,9	**6736**		
**6767**					71,8	**6738**		
**6768**					59,23	**6739**		
**6769**					52,6	**6740**		
**6776**					88,08	**6742**		
**6777**					62,28	**6783**		
**6786**					84,6	**8215**		0
**6787**					64,2	**8216**	41	0
**6788**					73,2	**8224**	5,7	0
**6789**					32,05	**8227**	2,4	0
**6792**					24,4	**8280**		
**6793**					62,3	**8281**	47,35	28,29
**6794**					58,6	**8282**	13,81	7,45
**6796**					81,6	**6725**		
**6797**					81	**6724**		
**6798**					3,9	**6800**		
**7801**					5,1	**6770**		
**7803**					37,26	**7852**	2,25	0,33
**7805**					87,7	**7842**	28,79	0,53
**7806**						**3613**		
**7809**						**7802**		
**7810**						**7819**		
**7813**						**6741**		
**7818**					75,7	**6778**		

**7822**					
**7826**					
**7828**					
**7829**					
**7831**	94,2	3,12			
**7832**					
**7833**					
**7835**					
**7837**	1,84	0,04			
**7844**	1,27	0			
**7846**	2	0,2			
**7849**	5,45	0,1			
**7854**	25,59	20,65			
**7858**	6,45	1,5			
**7859**	16	0,32			
**7863**	19,1	0,14			
**7866**	8,4	0,1		0,1	
**7868**	4,1				
**7869**	3,1	0,2	0,22	2,44	
**7872**	16,43	8	0,72	17,31	
**7873**	4,69	1,03	0,22	21,76	
**7874**	12	6,28	0,16	11,86	
**7876**	37,1	10,58	13,54	22,61	
**7880**	0,2	0,9	0	1,9	
**7883**	1,1	0	0	0,05	
**7884**	5,15	0,05	0,35	2,18	
**7889**	2,92	0,19	2,12	1,18	
**7891**	8,11	7,61	0,12	30,89	
**7892**	3,27	4,94	0,02	0,19	
**7893**	25,39	10,34	1,82		
**7899**	20,21	9,96	3,72	31,48	
**8042**	39,46	6,37	5,68	26,68	87,9
**8054**	1,45	19,22	17,17	72,28	10,18
**8056**	17,29	13,05	27,71	33,86	
**8057**	16,46	44,36	12,87	28,13	
**8061**	45,96	84,65		94,71	98,71
**8065**	46,74	80,71		70,43	75,52
**8066**	29,49	20,93		21,31	89,21
**8067**	16,16	28,27		2,05	19,86
**8068**	8,03	4,69		3,43	0,7
**8069**					
**7823**					
**7830**	9,8	0			
**7840**	14,04	3,2			
**7841**	7,3	0			
**7850**	6,6	0			
**7853**	9,5	0,29			
**7861**	2,54	0,03			
**7879**	0,21	0,36	1,69	1,69	
**8069**	8,05	2,24		3,41	0,05

**6799**		
**6784**		
**7855**	4,7	1,86
**7860**	9,24	0
**7862**	7,8	0,08
**7867**	17,6	
**6727**		
**6737**		
**6720**		
**3573**	21,23	21,41
**3574**	20,2	12,67
**3575**	12,85	3,86
**3576**	2,4	7,78
**3577**	3,05	19,6
**3578**		
**3579**	23,21	7,24
**3580**	18,68	7,92
**3581**	11,33	7,86
**3582**	21,08	10,93
**3612**		
**5831**	0,48	0,32
**5833**	0,33	1,19
**8225**	5,6	0
**8234**	3,2	0,99
**8283**	2,94	33,02
**6715**		

**8070**	64,3	35,52		34,03	86,55

CD8+ DE PD1+ İKON	CD19+ DA SLAM+ İKON	CD14+ CD46+ İKON	DNA NİKON	CD4+DE CD4+ SLAM+ NİKON	CD8+DE CD8+ SLAM+ NİKON	CD8+ DE PD1+ NİKON	CD19+ DA SLAM+ NİKON	CD14+ CD46+ NİKON
23,4	56,62	33,61	**6759**					48,34
7,67	66,19	57,55	**6779**					82,83
2,44	14,63	92,65	**6780**					78,6
2,25	27,26	56,76	**6781**					75,52
			**6782**					76,74
			**6785**					68,68
2,19	34,84	0,91	**7864**	4	1,42			
		72,89	**7870**	7,3	0,9	0,2	1,2	
		62	**8060**	68,24	*81.54**		79,22	92,55
		74,4	**8071**	99,05	*91.85**		63,56	84,99
			**7811**					
			**5832**	0,98	0,3	0,46	0,33	
	0,7		**5834**	4,93	0,66	0,1	0,66	
		35,7	**6743**					
		41,1	**6730**					84,84
			**7834**		1,1			
			**7851**	4,4	0			
		87,14	**7896**	0,67	5,19	0,48	2,05	
		84,2	**3559**	30,03	5,14	6,53	22,72	65,25
		76,5	**3560**	69,04	11,67	7,43	39,07	95,32
		71,3	**3565**					
		75,5	**3614**					
		54	**4344**					76,7
		69,3	**4379**					68,1
		79	**4382**					
		61,14	**6716**					91
		76,66	**6721**					73,2
		76,48	**6722**					67,2
			**7820**					56,5
			**7885**	5,73	0,07	0,01	0,37	
			**6728**					71,5
			**7827**					
			**6726**					32,5
			**7807**					
			**7815**					
		49	**7824**					
		63,5	**7825**					
			**7845**					
		26,89	**7812**					60,8
			**6744**					69,1
			**6733**					84,2
			**7847**	1,16	1,23			
			**7814**					
		60,7						
		80,41						

		11,2
		65,84
		30,9
		85,3
		51,4
2,81	54,16	
7,45	28,88	89,27
1,35	39,28	51,27
38,22	23,32	93,84
3,14	15,5	0,38
12,38		1,22
6,45	13,91	94,41
0,11	11,71	95,68
13,22	21,07	77,74
2,39	28,75	86,51
0,33	0,62	
0,8	1,03	
	37,71	
		93,9

SSPE DNA #	SSPE (-) ELISPOT SPON	SSPE MV-H ELISPOT	SSPE MV-C ELISPOT	SSPE MV-N2 ELISPOT	SSPE MV-M ELISPOT	SSPE MV-N1 ELISPOT	SSPE MV-POOL ELISPOT	IKON DNA #
**2315**								**3573**
**2325**	1	2	20	0	0	0	14	**3574**
**2379**								**3575**
**6707**								**3576**
**6712**								**3577**
**6719**								**3578**
**6729**								**3579**
**6734**								**3580**
**6735**								**3581**
**6750**								**3582**
**6751**								**3612**
**6752**								**3613**
**6753**								**3678**
**6754**								**3679**
**6760**								**3683**
**6762**								**3684**
**6763**								**3685**
**6764**								**5831**
**6765**								**5833**
**6766**								**6714**
**6767**								**6715**
**6768**								**6717**
**6769**								**6720**
**6776**								**6723**
**6777**								**6724**
**6786**								**6725**
**6787**	38	42	38	58	62	36	46	**6727**
**6788**								**6731**
**6789**								**6732**
**6792**	4	7	9	35	7	12	16	**6736**
**6793**	11	14	9	24	2	4	22	**6737**
**6794**	5	6	6	22	7	4	9	**6738**
**6796**								**6739**
**6797**	0	0	3	5	1	1	30	**6740**
**6798**								**6741**
**7801**	3	14	64	36	2	8		**6742**
**7803**	6	8	84	50	4	6	216	**6761**
**7805**	24	40	164	40	35	14	75	**6770**
**7806**	1	2	114	166	3	13	222	**6771**
**7809**								**6772**
**7810**	12	12	568	564	5	62	599	**6778**
**7813**	36	25	599	707	28	35	703	**6783**
**7818**	2	3	399	381	4	17	390	**6784**
**7822**								**6790**
**7823**								**6791**
**7826**	24	40	32	35	38	41	46	**6800**

**7828**								**7802**
**7829**								**7804**
**7830**								**7819**
**7831**								**7842**
**7832**								**7848**
**7833**								**7852**
**7835**	15	9	15	12	15	17		**7855**
**7837**								**7857**
**7840**								**7860**
**7841**								**7862**
**7844**								**7865**
**7846**								**7867**
**7849**								**7871**
**7850**								**8215**
**7853**								**8216**
**7854**								**8224**
**7856**								**8225**
**7858**								**8227**
**7859**								**8228**
**7861**								**8234**
**7863**								**8238**
**7866**								**8280**
**7868**								**8281**
**7869**								**8282**
**7872**	1	14	2	1	3	1	250	**8283**
**7873**	0	13	1	0	1	1	250	
**7874**	1	3	1	1	0	1	350	
**7876**								
**7879**	5	65	4	2	2	2	109	
**7880**	1	53	1	1	1	1	120	
**7883**								
**7884**	1	44	0	2	2	1	114	
**7886**								
**7888**	4	15	12	9	5	8	111	
**7889**	7	57	24	14	42	28	163	
**7891**								
**7892**	35	20	45	35	25	23	55	
**7893**	2	3	9	4	5	4	20	
**7899**								
**8042**	5	3	2	1	3	3	20	
**8054**								
**8056**								
**8057**								
**8061**								
**8065**								
**8066**								
**8067**								
**8068**								
**8069**								
**8069**								

8070
8070

IKON (-) ELISPOT SPON	IKON MV-H ELISPOT	IKON MV-C ELISPOT	IKON MV-N2 ELISPOT	IKON MV-M ELISPOT	IKON MV-N1 ELISPOT	IKON MV-POOL ELISPOT	NIKON DNA #	NIKON (-) ELISPOT SPON
0	0	0	0	0	0	0	**3559**	5
0	0	0	0	0	0	0	**3560**	2
1	1	1	0	0	1	2	**3565**	5
1	0	1	1	0	1	251	**3614**	4
0	1	0	0	0	1	0	**4344**	
0	0	1	0	1	1	2	**4379**	5
							**4382**	1
							**5832**	
							**5834**	
							**6716**	
1	1	0	0	0	0	0	**6721**	
3	1	0	1	0	0	0	**6722**	
							**6726**	
							**6728**	
1	1	1	1	2	1	4	**6730**	
							**6733**	
							**6743**	
							**6744**	
							**6759**	
							**6779**	
							**6780**	
							**6781**	
							**6782**	38
							**6785**	
							**6799**	6
							**7807**	
							**7811**	28
							**7812**	
							**7814**	13
							**7815**	10
							**7820**	6
							**7824**	
							**7825**	
							**7827**	
							**7834**	10
							**7845**	
							**7847**	
							**7851**	
							**7864**	
							**7870**	1
							**7885**	20
							**7896**	
							**8060**	
							**8071**	
33	162	840	536	17	13	1111		

7	2	15		36		26
1	4	357	24	1	0	79
1	14	1	1	0	1	105

NIKON MV-H ELISPOT	NIKON MV-C ELISPOT	NIKON MV-N2 ELISPOT	NIKON MV-M ELISPOT	NIKON MV-N1 ELISPOT	NIKON MV-POOL ELISPOT
3	2	1	3	3	20
3	2	3	2	7	9
5	6	4	6	8	10
1	1	0	0	0	0
15	21	10	14	5	23
2	34		7	1	3
42	38	58	62	36	46
5	42	11	3	3	14
27	342	376	22	3	402
7	54	342	10	6	315
20	142	279	76	35	833
9	6	285	4	6	140
1	2	2	2	11	6
1	1	0	1	1	250
16	14	15	12	17	62
