# Supplementary material for: Immune alterations in subacute sclerosing panencephalitis reflect an incompetent response to eliminate the measles virus
Source: PLoS One. 2021 Jan 7;16(1):e0245077. doi: 10.1371/journal.pone.0245077 (PMC7790413; doi:10.1371/journal.pone.0245077)
Supplement: Attachment — Submitted filename: Plos review.docx [file pone.0245077.s006.docx]

In this study, authors aimed to test the cytokine profile of lymphocytes and monocytes obtained from the blood of SSPE patients to gain more insight into the immunopathogenesis of disease. SSPE is a latent brain infection that occurs many years after measles infection and it is a fatal disease. It is an important health problem in some of the developing countries. It is relatively frequent in Turkey, and a high number of SSPE patients were tested in this study. As SSPE is very rare, this study has a potential to contribute to the related literature. However, some points need to be cleared:

Minor revisions:

- Exclusion criteria for all groups should be mentioned.
- How would the authors interpret the finding that IL10 secretion is lower compared to controls? Is this a result of the latent brain infection, or is this more like a risk factor for the disease as a result of genetic factors etc? A comment on that in the discussion would be useful.
- What is the possible functional outcome of the finding that IL12 is decreased in monocytes? A comment on that in the discussion would be useful.
- The authors stated that “In SSPE patients, the lower frequencies ofCD46+ monocytes compared with NICON group in this study also implicated a related interaction of the virus with these cells.” However, SSPE is known as a latent brain infection and active involvement of monocytes in the periphery would be a surprising finding which may refer to ongoing MV activity in peripheral organs. Can the authors make this point clearer in discussion?
- In the conclusion, authors state that their findings pointed at an attenuated inflammatory pattern at a chronic phase of SSPE. Considering that SSPE is a brain-restricted latent infection, what may be the reason behind the attenuated inflammatory pattern seen in the peripheral blood cells? Discussion of this point would be useful.
