# Supplementary material for: Immune alterations in subacute sclerosing panencephalitis reflect an incompetent response to eliminate the measles virus
Source: PLoS One. 2021 Jan 7;16(1):e0245077. doi: 10.1371/journal.pone.0245077 (PMC7790413; doi:10.1371/journal.pone.0245077)
Supplement: Attachment — Submitted filename: RtR-281020.docx [file pone.0245077.s007.docx]

Responses to Reviewers

Reviewer #1: The authors investigated the immune response in patients with SSPE in comparison to two well age-matched control groups consisting of inflammatory (ICON) and non-inflammatory (NICON) diseases. Taking into account that SSPE is a rare disease the number of patients is very high.
The authors used established methods (FACS, ELISA, Elispot assays, 3H thymidine incorporation). PBMCs of patients and controls were investigated ex vivo or after different stimulations in vitro – unspecific (antibodies, SAC) or MeV specific (viral peptides).
The results demonstrate, that PBMC of SSPE patients produced lower levels of IL-10 and IFN-g after stimulation with anti-CD3 and anti CD28, but were inducible to produce IL-2. After SAC stimulation PBMC of SSPE patients showed reduced IL-12p70 production and CD 14+ monocytes demonstrate lower CD46 surface expression. In Elispot assays spontaneous IFN-g production and antigen stimulated IFN-g production was elevated in SSPE patients and NICON compared with ICON.
The authors concluded, that T cells of SSPE patients demonstrate an altered immune response that is not sufficient to eliminate the virus. In monocytes reduced IL-12 production and CD46 surface expression implicate the effect of CD46 binding in SSPE similar to MeV infection.

Major points:
1. The authors report that in a recent epidemiological study of Istanbul a girl dominance was found. In their SSPE cohort there is a dominance of boys. Is this just by chance? From which part of the country or from which country were the patients recruited?

As pointed out by the reviewer, in our epidemiological study, the distribution of sexes was in favor of the women, whereas our cohort was not in agreement with this observation. The reason for this discrepancy may be related to the natures of the studies. The epidemiology was planned for the determination the incidence in Istanbul area in two years, whereas in our study the patients were collected throughout 14 years in the same area. Some information about the referral system of the patients is also added to the material and methods section.

2. Different clinical stages of SSPS are known. For reference see e.g. Jabbour J, et al., SSPE-clinical staging, course, and frequency. Arch Neurol. 1975;32(7):493–494. 24 or Gutierrez J, et al., Dev Med Child Neurol. 2010 Oct;52(10):901-7. It would be interesting to know in which clinical state the patients had been at the time of blood sampling.

We agree with the reviewer. Unfortunately the information related to the clinical stages of the patients was only available in a small number of patients (n: 44). As the patients were referred from several centers, we could not access their files later on. Patients with known stages were not representing the whole cohort, so that we did not include this information.

3. How do the authors interpret the reduction of T cells in SSPE samples?

As pointed at by the reviewer, a reduction of T cells in SSPE samples was observed in our previous study (reference 19) and in other studies (reference 30) as well. This observation may be related to the lymphopenia caused by MeV during the infection. MeV infected monocytes induce apoptosis in uninfected T cells which also may contribute to the pathogenesis of MeV-induced immunosuppression (reference 31). We have added this issue to the discussion.

4. The authors report “Monocyte stimulation with SAC”. In the legend of Fig. 4 they report “… SAC stimulated PBMC…”. If they don´t select monocytes before stimulation, the headline of this section and the legend of figure 4 should be modified. In addition, the authors should mention that SAC does not stimulate only monocytes in their cell culture.

As the reviewer highlighted, the assumption that the reduced IL-12 production is from monocytes was based on the fact that SAC would not be able to induce IL-12 by any other cells type in PBMC. The only differentially produced cytokine induced by SAC was IL-12, so that the monocytes have been accounted for this result. But we agree that the finding is based on an indirect finding and is an interpretation of the data. We have changed the text accordingly.

5. At the beginning of the discussion the authors claim “… no evidence was found for immunosuppressive mechanisms as a determining factor in SSPE development.” The authors may explain more detailed the reasons for this statement or omit it.

This sentence was meant as a general evaluation of the study and emphasized that the disease was not accompanied by an immunosuppression as observed in measles infection. We have changed this section now accordingly.

6. The authors report a reduced production of IL-10 in the present study, but mention a production of IL-10 as in controls in their previous study. Do the authors have an explanation for this?

We agree with the reviewer that the results of two studies are not in accordance for the spontaneous IL-10 response of the patients. The control group in our previous study was consisted of healthy adults whereas the controls in this study are age-matched diseased controls. The variance between cytokine measurements may be related to this parameter.

7. IL-10 is a cytokine with strong immunosuppressive properties. However, there are also publications demonstrating immunostimulation by IL-10, e.g. Il-10 enhance the capacity of resting CD4+ lymphocytes to produce cytokines. The authors may discuss this aspect as well and not solely the immunosuppressive properties of IL-10.

We agree with the reviewer that IL-10 has pleiotropic roles. However, in infectious diseases, apparently it mainly acts as regulator in a context dependent manner. As the production of IL-10 was reduced in our patient group, we have interpreted these finding as a dysregulation of regulatory response and added a comment to the discussion.

8. In the section “Antigen-specific T cell stimulation” the authors report “Interestingly, Ifn-g responses to all peptides and peptide pool were reduced in ICON group compared to SSPE (…) and NICON groups (…) …”. The authors may comment on this finding in the discussion, especially as they show an IFN-g response in PBMCs of ICON after unspecific stimulation (Fig. 2).

We agree with the reviewer about this comment on the unexpected findings. We consider that the IFN-g response to CD3+CD28 in ICON group is produced by a broad range of T cells as the stimulation is not specific in this case. On the other hand, the peptide specific IFNγ production in ICON group may be compromised particularly for the specific responses.

9. In the discussion the authors report “…SLAM expression was relatively higher in all cell subgroups both in SSPE and other inflammatory diseases compared with donors without inflammation…”. Fig. 3 demonstrate significant differences only for B-cells. This statement has to be modified.

The differences of SLAM we observed on CD4, CD8 and CD19 + cells were not significantly different and the only increase was detected on B cells of ICON group. That is why we did not mention the activation state of PMBC in the results section and in the discussion as a comment only. Now we have omitted this statement from the discussion, as it was not based on significant differences.

10. The authors report that “… the donors have been difficult to assign to the respective groups.” Please explain the reasons.

As some of the diseases we included in the controls are not easily assignable to inflammatory or no-inflammatory subgroups. Due to the subtle inflammatory changes in these disease states, we were not sure to include these patients in non-inflammatory cases (X-linked adenoleucodystrophy, nesioblastosis). We have added the some of the diagnosis in these groups.

Minor points:
1. Determination of CD46 and SLAM should be illustrated with an example showing a FACS analysis of a patient and two controls, e.g. as supplementary figures.

FACS analysis of SLAM and CD46 staining is now added as supplementary figures as requested (S1 Fig and S3 Fig).

2. In the section “Antigen-specific T cell stimulation” there is a list of p-values. It is unclear to which peptide stimulation a given p-value belongs. The authors should present peptide stimulation and corresponding p-value in a supplementary table or omit the p-values in the text, as they are given in Fig. 6.

We have omitted the p values from the text.

3. The citation format of two references in the discussion does not comply with the journal style.

We have corrected them.

Reviewer #2: In this study, authors aimed to test the cytokine profile of lymphocytes and monocytes obtained from the blood of SSPE patients to gain more insight into the immunopathogenesis of disease. SSPE is a latent brain infection that occurs many years after measles infection and it is a fatal disease. It is an important health problem in some of the developing countries. It is relatively frequent in Turkey, and a high number of SSPE patients were tested in this study. As SSPE is very rare, this study has a potential to contribute to the related literature. However, some points need to be cleared:

Minor revisions:

1. Exclusion criteria for all groups should be mentioned.

Based on the diagnosis of the patients, we did not exclude any patients with SSPE in the study group. Only patients whose parents did not agree to consent were excluded. All other patients who were not diagnosed with SSPE were included in the control groups according to the disease with or without apparent inflammation. We added this information to the manuscript.

2. How would the authors interpret the finding that IL10 secretion is lower compared to controls? Is this a result of the latent brain infection, or is this more like a risk factor for the disease as a result of genetic factors etc? A comment on that in the discussion would be useful.

We have added a comment to the discussion related to lower IL-10 production in SSPE. We considered the reduced production of IL-10 in combination with reduced IFN-γ production as an inefficiency of effector functions of T cells. An exhausted state which we could not demonstrate would be the good explanation for that.

3. What is the possible functional outcome of the finding that IL12 is decreased in monocytes? A comment on that in the discussion would be useful.

As IL-12 is a key inducer of Th1 responses, the decreased activity of Th1 type cells reflected by the lower IFN-γ production could be the consequence of this finding. We have added a comment to the discussion.

4. The authors stated that “In SSPE patients, the lower frequencies ofCD46+ monocytes compared with NICON group in this study also implicated a related interaction of the virus with these cells.” However, SSPE is known as a latent brain infection and active involvement of monocytes in the periphery would be a surprising finding which may refer to ongoing MV activity in peripheral organs. Can the authors make this point clearer in discussion?

Although this explanation is not evidence based, we have added some speculative considerations to the discussion about these data. Unfortunately we do not have a good explanation for the development in SSPE in these terms.

5. In the conclusion, authors state that their findings pointed at an attenuated inflammatory pattern at a chronic phase of SSPE. Considering that SSPE is a brain-restricted latent infection, what may be the reason behind the attenuated inflammatory pattern seen in the peripheral blood cells? Discussion of this point would be useful.

With the given data, SSPE is considered as a result of reactivation of a latent infection after many years. The RNA from the measles virus has been isolated in the brain, eyes, and spinal cord in patients with SSPE and persistence of MeV was confirmed in PBMC even after years. An immune response is generated against the virus and a strong antiviral immune response is induced as evidenced by the unusually high levels of antibody in serum and CSF. But it is not effective in eliminating virus or controlling replication in the CNS. During persistence, viral RNA in PBMCs and lymphoid tissue is detected in B, T lymphocytes and monocytes. Persistent RNA in lymphoid cells may contribute to immune response dysfunction by altering the ability of cells to proliferate in response to immune signaling (reference 39).
