# Supplementary material for: Immune alterations in subacute sclerosing panencephalitis reflect an incompetent response to eliminate the measles virus
Source: PLoS One. 2021 Jan 7;16(1):e0245077. doi: 10.1371/journal.pone.0245077 (PMC7790413; doi:10.1371/journal.pone.0245077)
Supplement: Attachment — Submitted filename: RtR-151220.docx [file pone.0245077.s008.docx]

PONE-D-20-23751R2
Responses to the reviewer

Point #1: If there were no exclusion criteria, were there any patients or controls who have a concomitant acute/chronic infection (other than SSPE), or autoimmune disorder at the time of blood sampling?

There were no SSPE patients with other disorders according to the assessment of the following clinician. We have added a remark about the problem of data acquisition from the patients in the methods section.

Point #4: The lower frequencies of CD46+ monocytes compared with NICON group in the current study is an interesting finding and as the authors state “may implicate a related interaction of the virus with these cells.” Can the authors provide data regarding the presence or absence of MeV RNA in monocytes from SSPE patients?

In this study, although we intended to identify the virus in the patient material in the beginning, we were not able to assess the MeV RNA in the samples of SSPE patients. However, the presence of viral RNA has been documented in monocytes during acute measles infection (Reference 39). MeV infection has been also shown to suppress IL-12 secretion from monocytes (Reference 15). Assuming that the virus used CD46 to enter the monocytes, we claimed that the decreased IL-12 production and CD46 expression could fit well with this picture. Unfortunately, there is no evidence of the viral persistence in the peripheral blood cells, including monocytes, shown formally in SSPE. We have changed the section accordingly.

Point #5:
In response to the point #5, the authors commented that “With the given data, SSPE is considered as a result of reactivation of a latent infection after many years. The RNA from the measles virus has been isolated in the brain, eyes, and spinal cord in patients with SSPE and persistence of MeV was confirmed in PBMC even after years…. During persistence, viral RNA in PBMCs and lymphoid tissue is detected in B, T lymphocytes and monocytes. Persistent RNA in lymphoid cells may contribute to immune response dysfunction by altering the ability of cells to proliferate in response to immune signaling…”
Reference #39 given as the source for this information is a review article and from its references it is seen that persistence of MeV was shown in Macaque Monkeys, HIV-infected children, or shortly after acute infection, but not after many years, or in children with SSPE. However, this is an important and interesting point. Could the authors provide more specific literature, or better, could they provide data regarding the presence or absence of MeV RNA in lymphocytes and also in monocytes, in SSPE children vs controls?

The hypothesis that “the persistence of MeV RNA may contribute to the late development of the slowly progressive disease subacute sclerosing panencephalitis in children infected at a young age” (reference 22) has been proposed by the authors studying measles infection. We also found this approach somehow suitable to our findings. As pointed at by the reviewer, there are no data on the presence of the viral RNA in any peripheral cell or tissue in SSPE. However, based on the proposed similarities between the monkey model and the humans in terms of measles (*Measles virus infection in rhesus macaques: Altered immune responses and comparison of the virulence of six different virus strains. J Infect Dis. 1999;180:950–958*), we assumed that this explanation can also be applied to our data. Now we have changed the section accordingly. Unfortunately, we do not have any data about the presence of MeV RNA in any cell group of SSPE patients in this study group.

In addition, the authors stated that “An immune response is generated against the virus and a strong antiviral immune response is induced as evidenced by the unusually high levels of antibody in serum and CSF. But it is not effective in eliminating virus or controlling replication in the CNS.”
This is again an interesting and important point. According to the authors’ hypothesis, persistence of MeV and slow replication of the virus in the peripheral lymphoid organs and blood can be a source for the latent brain infection. And generation of a higher titer of anti-measles antibodies would show the presence of the viral persistence, but would be ineffective to eliminate the virus. Can the authors provide the specific literature showing the higher titer of anti-measles IgG in SSPE patients compared to controls? Or better, could they provide the data as an addition to the current study?

We thank the reviewer for raising this point. Unfortunately, we were not precise in our wording: what we know about the antibody response in SSPE is that anti-MeV antibodies are present in the CSF of patients and this is utilized even as a diagnostic finding for SSPE (Reference 16 and 17). The clinical diagnosis of SSPE is confirmed by the finding of increased levels of anti-measles virus anti- body in serum and CSF with an elevated CSF-to- serum ratio in antibody level. Actually, most of the time the antibodies are also detected in the plasma. In our own experience, we have screened the oligoclonal IgG bands in 108 of these study patients. The positivity of IgG bands in the CSF was 100% with a few atypical patterns and all OCB positive patients had also anti-MeV antibodies in the CSF. In 82.4 % of the patients, serum samples also contained accompanying and presumably leaked IgG bands (pattern 3 in 79.6%, pattern 4 in 2.8%). Although these antibodies were not tested for their specificity for MeV, there are data supporting that these antibodies are MeV specific. Our interpretation of the peripheral anti-viral antibody was based on the oligoclonal bands in the patients. The findings only allow to explain the immune response in periphery induced by leaked antigens and antibodies from the CNS. We have also changed the section in the discussion accordingly.
